# Redefining drug discovery: AI-driven strategies for efficient cancer therapeutics

**DOI:** 10.3389/fonc.2026.1907591

**Published:** 2026-10-05

**Authors:** Diptanu De, Sohini Chakraborty, Satarupa Banerjee

**Affiliations:** 1 Department of Biotechnology, School of Biosciences and Technology Vellore Institute of Technology, Vellore, India; 2 Department of Biotechnology, School of Applied Sciences, REVA University, Bengaluru, India

**Keywords:** artificial intelligence, clinical therapeutics, data digitization, drug discovery, healthcare, machine learning

## Abstract

The practice of artificial intelligence has recently expanded several social spheres, with the pharmaceutical domain leading the way. Given that AI can facilitate rational drug design, it is plausible to envision its involvement in developing pharmaceutical products from the laboratory to the patient’s bedside. AI recognizes hit and lead compounds, followed by validation of therapeutic targets and optimization of molecular design. Artificial intelligence significantly impacts drug discovery and development processes, including target identification, molecule design, drug-drug interactions, drug repurposing, optimization of clinical trials, drug toxicity prediction, drug formulation, and delivery. AI-assisted approaches are progressively being employed for identification and prioritization of targets, biomarker discovery and virtual screening as well as prediction for the interaction of protein and ligand and their molecular properties followed by ADMET screening, molecular design and repurposing to be suited for clinical trial optimization. The significance of automation will be boosted by adopting the most recent artificial intelligence-based technologies, reducing the time it takes for drugs to be available in the market with improved quality, production process safety, and resource utilization. The application of artificial intelligence may not only aid in rapid decision-making but can also enhance the process of incorporating the newly discovered drug into the optimum dosage form, which leads to faster manufacturing of higher-quality drugs and ensures consistency among batches. The potential of artificial intelligence in drug discovery and development, has been explored in this review, encircling several stages from identification of target to its clinical applications, followed by drug development approaches encompassing the future of artificial intelligence in the pharmaceutical sector, along with crosstalk on the tools and tactics used to enforce artificial intelligence, current difficulties, and respective solutions

## 1 Introduction - the concepts of artificial intelligence and its role in cancer drug discovery:

AI can imitate human intellect using various cutting-edge tools and network-based approaches (1). AI allows us to eliminate tangible human abilities from the training, logistics, problem-solving, perception, and decision-making processes. Automation of chores, prediction making, and complicated problem-solving are all possible with the help of AI systems that mimic human intelligence using algorithms, data, and processing power. Machine learning (ML), deep learning (DL), computer vision, and other AI-related technologies are only a few examples. The ML system used patterns in the input data to train a predictive model. Classical machine learning algorithms form the foundation to explore the potential and restrictions of various AI models. Using a reverse-propagation approach, DL can uncover complex structures in large-scale datasets. Healthcare is an industry with increased use of AI, which is developing quickly and influencing the direction of automation (2) (**Figure 1**). Data digitization in the clinical industry has increased over the last two decades. To address challenging clinical issues, it is difficult to obtain, analyze, and implement this information as a result of digitalization (3). This results in increased AI technology owing to its ability to manage large-scale data with improved automation (4). A technology-based system called AI can imitate human cognitive abilities using various advanced tools and networks (1). Rather than replacing researchers, AI is increasingly being positioned as an augmented-intelligence technology that supports human decision-making, hypothesis generation and experimental prioritization. AI enhances the skills of human researchers, frequently exceeding other approaches in areas such as drug-target interaction modelling and therapeutic repositioning.

**Figure 1:**
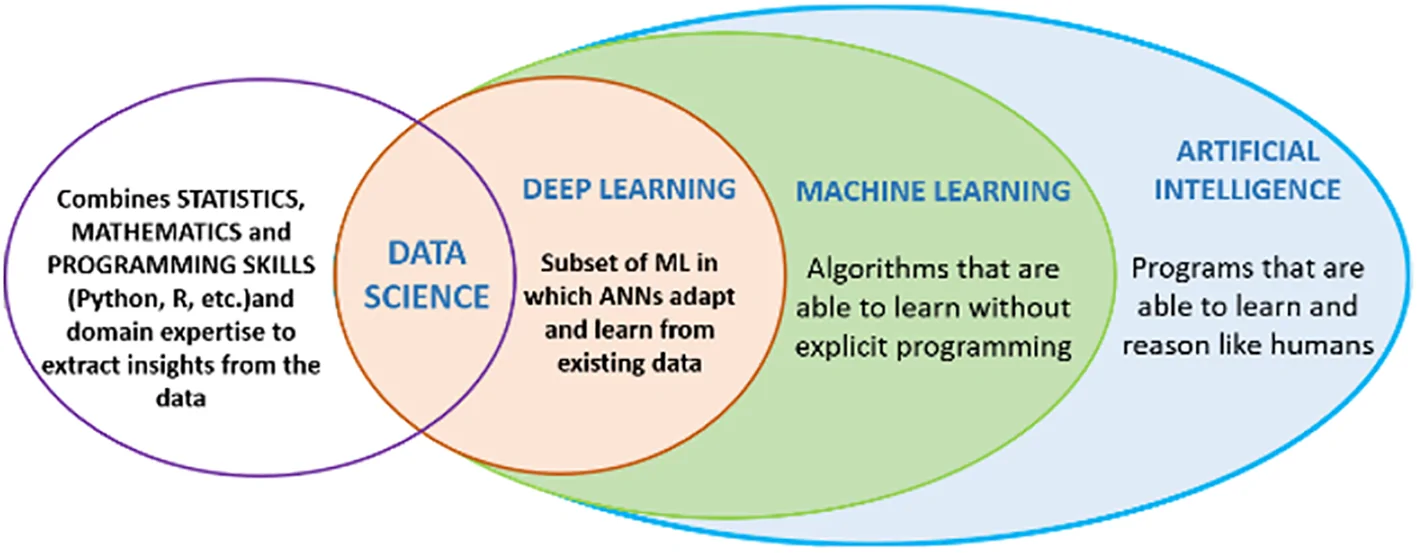
Hierarchical relationship between artificial intelligence, machine learning, deep learning and emerging foundation-model architectures in cancer drug discovery.

Cancer drug discovery delivers a particularly challenging application of AI because therapeutic response is dependent on interactions between genetic alterations, epigenetic states, signalling networks, tumour microenvironment, immune context, metabolic architecture and medical history. Accordingly, recognizing a statistically associated molecular feature is inadequate for establishing therapeutic significance. Translational AI systems must differentiate biomarkers of disease association from targets that are causally essential for tumour maintenance and that remain therapeutically actionable *in vivo*. This distinction is particularly important in precision oncology, where interpatient and intratumor heterogeneity can cause apparently promising targets to exhibit highly variable clinical dependence.

When provided with relatively small structured and interpretable datasets, Classical machine-learning methods are satisfactory, however for high-dimensional molecular, imaging or omics datasets with nonlinear relationships that are hard to encode manually, deep-learning models are more advantageous, where models like graph neural networks (GNNs), geometric deep-learning models as well as transformer-based architectures can operate easily on biological dataset consisting of 3D structures, or sequences (5, 6).

To fully realize the potential of AI drug discovery, a major bottleneck that must be eliminated is the availability of vast quantities of high-quality data for model training. There are dozens or hundreds of AI/ML approaches for drug discovery (7). For instance, various branches of ML (like as supervised, unsupervised learning and reinforcement learning) exist, along with numerous algorithmic approaches within these branches (like linear regression, neural networks, and support vector machines), as well as a variety of application areas (such as patient stratification, target identification (TID), and generative chemistry), all of which can be combined (7).

The appearance of generative AI has further transformed the role of computational models as they can predict molecular structures and optimize them against multiple objectives like potency, selectivity, physicochemical attributes, synthetic accessibility and ADMET properties rather than predicting predefined molecules. Foundation models can learn general representations from very large datasets to specific downstream applications. For instance, a recent review identified over 200 biological or drug-discovery foundation models spanning over target discovery, molecular optimization and preclinical applications (8).

## 2 Evolution of AI in drug discovery

Before AI-mediated healthcare facilities, training such models needs to be performed utilizing clinical data such as screening, diagnosis, and treatment assignment. This allows AI models to identify subject groups with similar feature patterns, leading to anticipated outcomes (9).

**Figure 2** portrays a roadmap of the progress of artificial intelligence in drug discovery and development with the steady implementation of artificial intelligence in pharma research. Starting in the 1950s, the very start of the concept of artificial intelligence in scientific research have been reported. Preliminary AI methods were being researched in 1971, to support drug research but in a very imperfect way due to the limitations in computational capacity of that time. By the 2000s, AI began to play a more momentous role, with enhancements in domains expanding from machine learning, computational chemistry, to molecular modelling, significantly empowering processes of drug discovery. In 2006-07, AI-based approaches started to take off, refining predictive modelling and bioinformatics procedures. The 2010s observed a steep rise in the use of deep learning-based approaches, specifically in the process of drug screening, protein structure, and toxicity prediction. The breakthroughs happened between 2018 and 2020 where deep learning models, graph neural networks, and AI-based clinical monitoring transformed the pharma industry (10). Lastly, by 2022-23, AI further expanded, with enhanced algorithms permitting smart drug design, personalized medicine, as well as real-time data analysis for drug formulation and synthesis (11). This timeline graphically shows how AI has progressively transmuted the drug discovery and development space over the decades.

**Figure 2:**
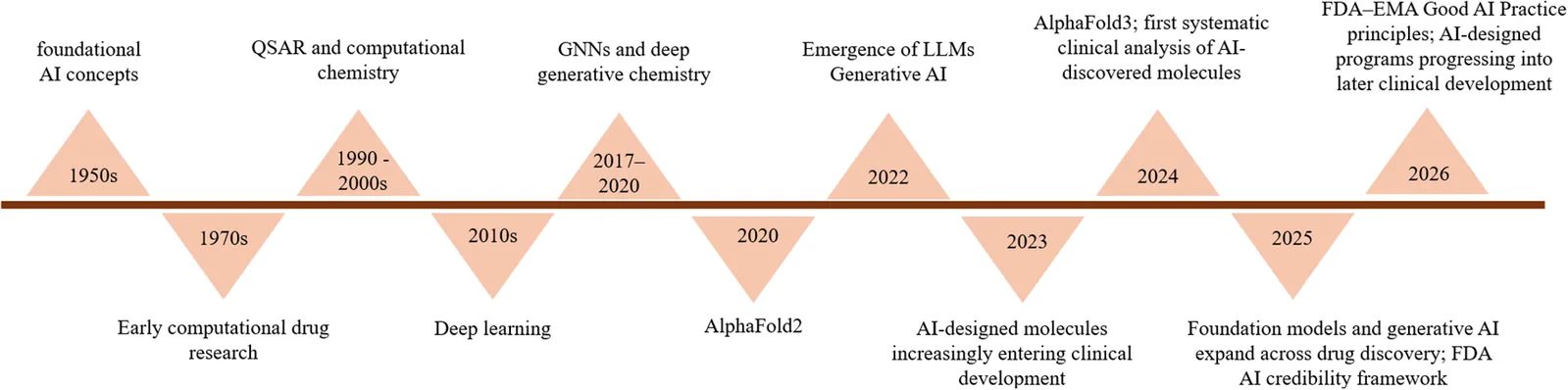
Evolution from early AI to foundation-model-enabled cancer drug discovery, 1950–2020.

Augmented intelligence: AI will be developed and used to complement rather than replace human intelligence, according to the American Medical Association (AMA), which recently referred to the use of AI in healthcare as “augmented intelligence” (12). The AMA encourages collaboration between humans and machines (**Figure 3**), a vital aspect of AI in the healthcare industry. Such a collaboration does not imply that the machine can be operated independently (13). AI automation is feasible for tasks in which either machine outperforms humans (cancer screening, diabetic retinopathy, and cardiac morbidities), where errors rarely lead to significant alteration of outcomes (risk-prone patient population), or machine-mediated procedures due to the balance in human-machine collaboration (types of care we value Vs levels of automation) is crucial in the AI-mediated healthcare.

**Figure 3:**
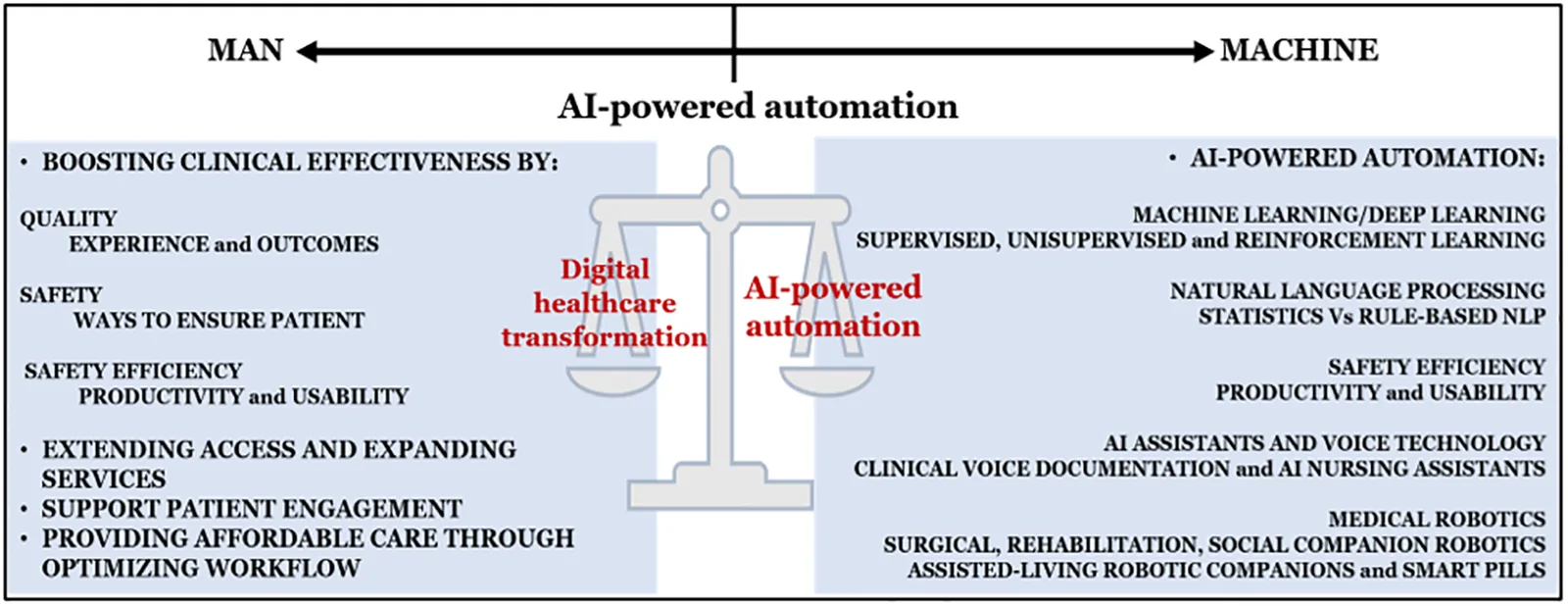
Human–AI–experiment feedback loop in cancer drug discovery.

Learnability: An essential element of AI and ML algorithms is “learnability”, which enables autonomous training of such models from existing data to improve model efficiency over time without explicit programming.

A dataset and predetermined labelled results are used as the input and output, respectively, in supervised learning. Patterns are then found that link the results and the input to make predictions (14). Medical imaging is utilized to diagnose the subclass and acuteness of cancer tumours using predictive analytics within a continuous output (Electronic Health Record predictions about the relapse, prognosis, and survival rates in a disease) (15). Unsupervised learning determines the data structure required to generate input-based predictions.

Unsupervised learning is closer to “true AI” due to its ability to autonomously “train” without human output labelling (16). On the other hand, unsupervised learning is more error-prone without human instruction because it might make predictions based on unimportant data properties. At times, semi-supervised learning combines supervised and unsupervised learning by using unlabelled data in a significant ratio: a very small amount of training benefits both training methods.

With rewards and mistakes as feedback to direct training, reinforcement learning (RL) is a more autonomous training method that enables machines to act and interact with the environment (17). It can be employed when an agent needs to continually interact with the environment and modify its activities (optimize treatment plans and robot-assisted surgery) based on feedback from the environment. Some healthcare supply and demand issues may be resolved using AI in the healthcare industry. AI, combined with technological advancements (internet, computational techniques, and data privacy) and multimodal data (genomics, economic, demographic, clinical, and phenotypic), helps healthcare and technology fundamentally transform models of healthcare delivery through AI-mediated approaches (17).

**Figure 4** provides a comprehensive overview of the diverse applications of artificial intelligence (AI) across the drug development pipeline. At its core, AI serves as an enabling technology that supports multiple stages of pharmaceutical research and development, from drug discovery to manufacturing and quality assurance.

**Figure 4:**
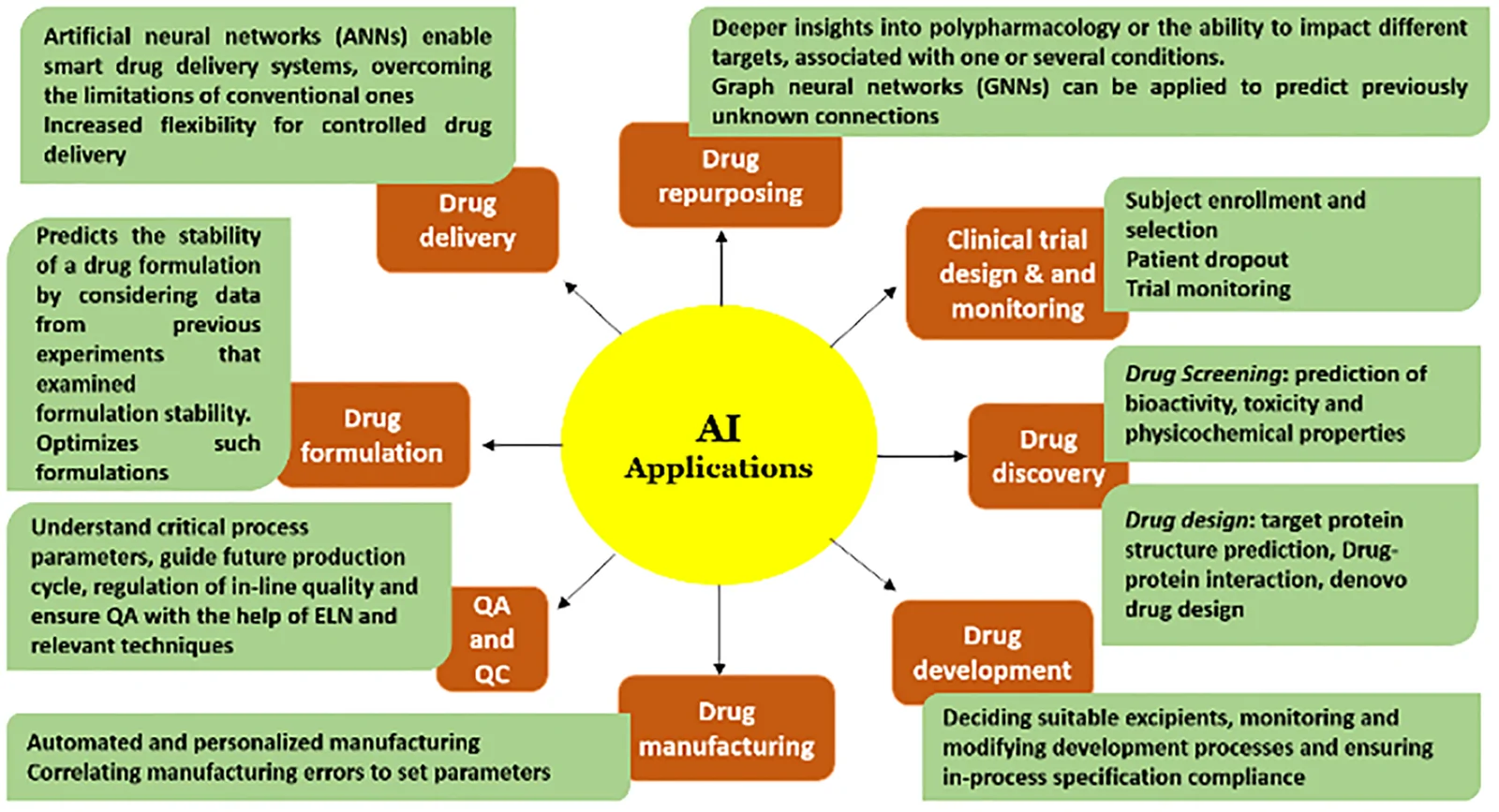
AI across the cancer drug-discovery and development continuum.

In the drug discovery phase, AI accelerates the identification of potential drug candidates by facilitating virtual screening and predicting biological activity, toxicity, and physicochemical properties. These capabilities reduce both the time and cost associated with conventional screening methods. AI also plays a critical role in drug design by predicting target protein structures, modelling drug–target interactions, and enabling the *de novo* design of novel therapeutic compounds, thereby streamlining the development of promising drug candidates (18).

Beyond discovery, AI has transformed drug development by optimizing formulation processes, guiding excipient selection, and ensuring pharmaceutical compliance. In clinical trials, AI enhances patient recruitment and stratification, predicts participant dropout rates, and improves overall trial design and efficiency through the analysis of large and complex datasets (19). Additionally, AI contributes significantly to drug repurposing, where machine learning approaches, including graph neural networks (GNNs), identify novel therapeutic indications for existing drugs by uncovering previously unrecognized relationships between diseases, biological targets, and chemical compounds (20).

AI also plays an important role in drug formulation, where predictive models trained on historical experimental data optimize formulation parameters and assess drug stability, thereby supporting the development of safe, stable, and effective pharmaceutical products. In drug delivery, artificial neural networks (ANNs) facilitate the design of intelligent delivery systems by optimizing formulation characteristics, improving targeting accuracy, and controlling drug release profiles, ultimately enhancing therapeutic outcomes (21).

In pharmaceutical manufacturing, AI-driven automation enables process optimization, personalized production strategies, real-time detection of manufacturing deviations, and continuous monitoring of process variables to ensure product consistency (22). Furthermore, AI strengthens quality assurance (QA) and quality control (QC) by supporting process optimization, enabling real-time in-line quality monitoring, and integrating with electronic laboratory notebooks (ELNs) and other digital systems. These capabilities enhance regulatory compliance, operational efficiency, and the overall reliability of pharmaceutical manufacturing processes (23).

In contrast to the solitary application of a single imaging technology, multimodal imaging offers more spatial or molecular information and strives for complementarity, rather than redundancy (24). Multimodal imaging enables quick progress in drug discovery and development initiatives and helps decision-makers make well-informed choices; therefore, many substances can be eliminated early owing to their unfavourable potency, pharmacokinetics, or safety characteristics. This early prioritization of compounds with appropriate properties is anticipated to significantly lower expenses in later phases (25).

Industry 4.0 in drug designing. Industry 4.0, also known as the Fourth Industrial Revolution, refers to the integration of advanced digital technologies into manufacturing and industrial processes through the convergence of digital, physical, and biological systems (26). This transformation has significantly influenced pharmaceutical research and development (R&D) by enabling faster, more efficient, and increasingly personalized drug discovery and development processes (27).

A fundamental feature of Industry 4.0 is the integration of artificial intelligence (AI) with technologies such as big data analytics, cloud computing, robotics, automation, simulation, and the Internet of Things (IoT). The extensive use of big data and real-time analytics enables AI to process and interpret vast volumes of information generated from genomics, clinical trials, electronic health records (EHRs), and drug–drug interaction studies (28). By leveraging these datasets, AI can identify novel drug targets, predict molecular properties, optimize drug design, and forecast clinical outcomes, thereby accelerating decision-making throughout the drug development pipeline. Furthermore, AI-driven analysis of patient-specific genomic and clinical data facilitates the identification of disease biomarkers, supporting the development of precision medicines tailored to individual patient characteristics (29).

Robotics and automation, which are central components of Industry 4.0, have also transformed pharmaceutical research. High-throughput screening (HTS) systems integrated with AI algorithms can rapidly evaluate thousands of chemical compounds, identify promising drug candidates, and prioritize them for further development. This significantly reduces the time, cost, and labour associated with traditional screening approaches while improving the efficiency and accuracy of candidate selection (30).

Cloud computing further strengthens Industry 4.0 by enabling secure data storage, large-scale data sharing, and real-time collaboration among researchers across different geographical locations. These cloud-based platforms allow AI models to be trained using heterogeneous datasets from multiple sources, improving their predictive performance and facilitating the discovery of complex biological relationships that may not be evident from isolated datasets. Additionally, cloud computing supports AI-driven simulations of drug interactions, disease progression, and physiological responses, providing the foundation for virtual clinical trials that reduce reliance on costly and time-consuming experimental studies (31).

Another key aspect of Industry 4.0 is the use of digital twins, which are virtual representations of biological systems or patients that enable researchers to simulate drug responses under various physiological conditions. By integrating real-time patient information, including genetic, environmental, and lifestyle factors, digital twins combined with AI facilitate personalized treatment planning and support the development of customized therapeutic strategies (32). These technologies enable researchers to predict treatment outcomes more accurately while minimizing experimental risks and costs.

Beyond drug discovery, Industry 4.0 technologies have revolutionized pharmaceutical manufacturing and quality management. AI-powered predictive analytics can utilize data from clinical trials, genomic sequencing, manufacturing processes, and electronic health records to optimize production workflows and improve decision-making. Automated quality control systems continuously monitor manufacturing parameters, detect deviations in real time, and ensure compliance with Good Manufacturing Practices (GMP), thereby reducing human error and improving product consistency (33). Moreover, AI-based surveillance systems support regulatory compliance by automating documentation, monitoring manufacturing operations, and facilitating continuous quality assurance. Collectively, the integration of AI within the Industry 4.0 framework has transformed pharmaceutical development into a more intelligent, data-driven, and patient-centered process, improving efficiency, product quality, and the likelihood of successful therapeutic outcomes.

## 3 Landscape of AI methodologies in cancer drug discovery

AI based approaches in cancer drug discovery differ considerably in their inputs, data requirements, interpretability computational cost and translational utility. For small structured datasets Classical machine-learning approaches like random forests, support-vector machines and gradient-boosting algorithms are competitively advantageous with lower computational requirements, robustness and more interpretability, however, their predictive performance is built strongly on how well the descriptors are manually engineered and not on complex molecular relationships (34).

Deep neural networks are primarily useful for analysis of images, sequences, molecular properties and multimodal biological data as they can learn nonlinear relationships from high-dimensional data and generally need larger training datasets and are sensitive to overfitting, data leakage and distribution shift. Small atoms as nodes and chemical bonds with edges can be represented by GNNs, where message-passing architectures learn local chemical environments and does not require relevant structural features to be explicitly encoded as fingerprints. AttentiveFP allows model to emphasize chemically informative structures via attention mechanism of GNN. Studies have demonstrated that GNNs can outpace conventional descriptor-based approaches for some ADME tasks, but their efficiency depends on the dataset size, chemical-space similarity and validation strategy (34).

Recent cancer-specific studies highlight the growing role of GNNs in therapeutic discovery. In a 2026 study of triple-negative breast cancer, multiple GNN architectures, including AttentiveFP, GATv2, TransformerConv, GCN, GIN and GraphSAGE, were benchmarked for AKT1 inhibitor prediction using both random and scaffold-based data splits. Such studies are imperative because scaffold-based assessment delivers a more rigorous valuation of generalization to chemically novel compounds than random splitting alone (35).

Generative models like variational autoencoders, generative adversarial networks, reinforcement learning systems and transformer based molecular language models converse the traditional prediction example by producing candidate assemblies optimized toward specified objectives. Their primary benefit is exploration of chemical space; while their limitation is that computationally wanted molecules may still be unnaturally unreachable, biologically sedentary or pharmacologically risky. Thus, generative AI should be united to retrosynthesis, experimental fusion, biochemical analyses and reiterative medicinal-chemistry optimization.

Geometric deep-learning models rely on graph - based methods by including 3D coordinates, distances, angles and symmetry information and is predominantly applicable for structure-based drug design since protein - ligand recognition is essentially geometric. Recent geometric deep - learning based approaches have been used for prediction of molecular properties, ligand pose, binding sites and ligand generation (6, 36).

Foundation models and LLMs denote the latest layer of the AI ecosystem. Their asset is extensive depiction learning and multimodal data integration rather than dominance in every individual prediction duty. In cancer drug discovery, they may participate literature - derived knowledge, omics data, molecular structures, clinical records and experimental data, thus empowering hypothesis generation transversely conventionally detached stages of study. However, application of LLM - generated hypotheses in biomedical decision-making may lead to hallucination, provenance, reproducibility and validation (10, 37).

Various methods are generally considered in the context of ML, including the Random Forest (RF) Algorithm (38), Naive Bayesian Classification (NBC) (39), Multiple Linear Regression (MLR), Logistic Regression (LR) (40), Linear Discriminant Analysis (LDA), Probabilistic Neural Networks (PNN) (41), multilayer perceptron (MLP), and Support Vector Machine (SVM) (42). AI developments are especially applied as a DL approach for medication design to improve feature extraction and generalization capabilities. The convolutional neural network (CNN), recurrent neural network (RNN), auto-encoder, deep neural network (DNN) (42), and Radial Basis Function Network (RBFN) are considered different DL techniques (**Figure 5**).

**Figure 5:**
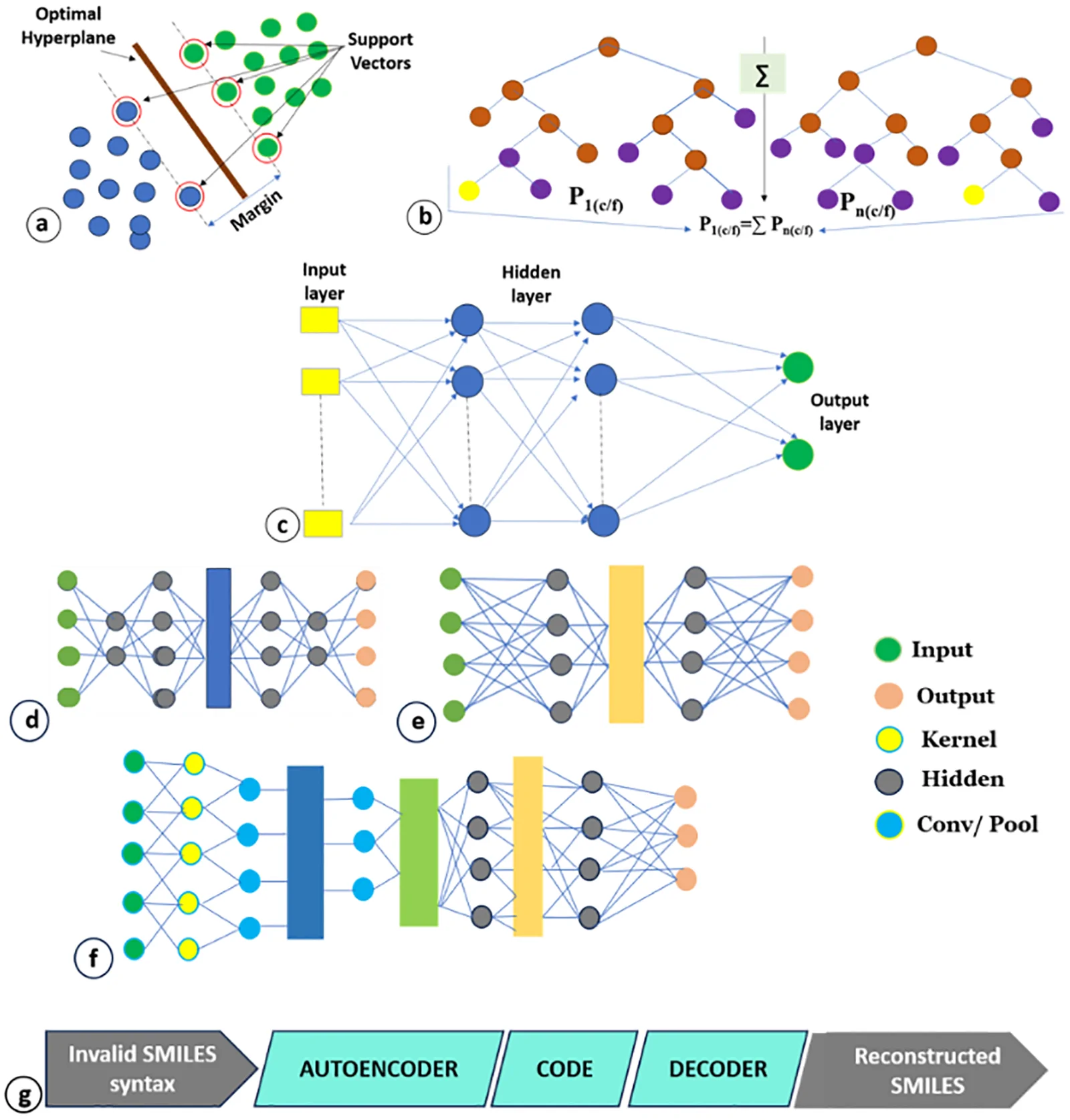
The concept of different approaches in AI. **(a)** Maximum-margin-hyperplane for an SVM trained with samples from two classes. Samples on the margin are support vectors. **(b)** Random Forest algorithm generated for data point decision tree to retrieve estimations for best outcomes via ‘voting’. **(c)** Multilayer Perception Architecture. **(d–f)** DL architectures. **(g)** Basic flow chart of grammar variational autoencoder for correcting SMILE syntax.

### 3.1 Target identification

TID is the process of discovering proteins or other compounds that can be targeted by medications to treat a certain condition. AI plays an increasingly significant role in this process. By analysing enormous databases of genomic, proteomic, and other biological data, AI can assist in the discovery of new therapeutic targets in ways like (41). Identification of targets in cancer is primarily suited to AI since oncogenic phenotypes arise from interactions among different phenomena like genomic alterations signalling networks, factors of tumour microenvironment, and medical histories. Apart from treating a statistically associated differentially expressed gene as a target, AI-backed discovery of targets combines multi - omics records, topological networks, genetic and functional data as well as clinical information to prioritize targets with disease relevance (41).

#### 3.1.1 Network-based strategies

AI can examine biological networks based on the shortest path, module/clique detection, and network centrality. For instance, AI can identify proteins differentially expressed in diseased cells or highly related to other proteins in the network. The shortest route algorithm is used to discover the shortest connection between two genes or proteins in the graphical depiction of a cellular network. One type of network dispersion method, module discovery algorithms, discovers communities of cancer genes in complicated networks by examining their topological structures. Node centrality is a metric of node relevance that can be used to intelligently identify significant nodes with important biological activities in network biology.

#### 3.1.2 ML strategies

To find prospective pharmacological targets, ML models, such as decision trees, can also be trained using AI. These models can be used to determine whether a specific protein is likely a therapeutic target after training. A decision tree is a supervised classification framework with three stages: feature selection, decision tree construction, and decision tree trimming (43). Non-graph ML models, such as RNN and DNN, and graph-based models, such as GCN, GAE, GAN, and DeepWalk, are used for TID. The steps involved in TID are depicted in **Figure 6**.

**Figure 6:**
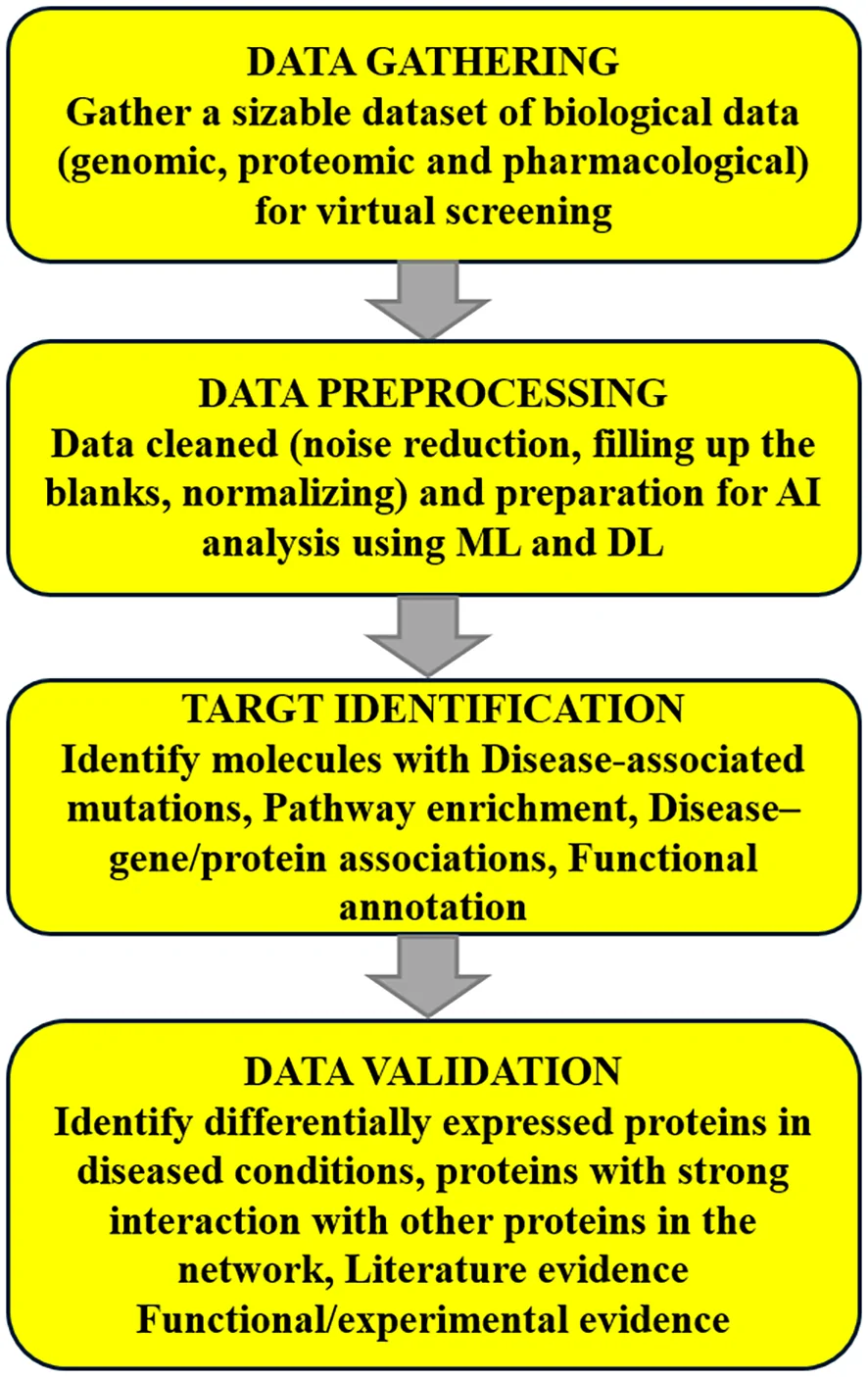
Sequential steps involved in AI assisted TID (target identification) in drug discovery.

There are some instances of recent developments in TID using AI. A group of scientists from the University of California, San Francisco, employed AI in 2022 to find a novel pancreatic cancer therapeutic target. Researchers employed an AI model to analyze a dataset of more than 10,000 tumour samples from patients with pancreatic cancer. The AI model discovered an intriguing therapeutic target as a protein named WEE1, a kinase involved in cell division and survival (44). A group of scientists from Insilco Medicine who employed AI in 2023 created a new medicine that suppresses WEE1, and they demonstrated in the lab that the drug was a novel therapeutic target for Alzheimer’s disease. Researchers have utilized an AI model to analyze a dataset of genomic and proteomic information from Alzheimer’s patients. The AI model recognized the LRRK2 protein as a potential therapeutic target. Protein transport and autophagy are two biological processes in which LRRK2 participates (45, 46).

Another notable instance of AI assisted discovery programme which targets the cyclin-dependent kinase 20 (CDK20) in hepatocellular carcinoma (HCC), which is based on target prioritization via the combination of PandaOmics along with protein structures predicted using AlphaFold as well as Chemistry42 generative chemistry platform which recognised CDK20 as a candidate target for HCC. The pipeline produced over 8900 candidate structures of which seven were prioritized for synthesis and biological evaluation, where one of them exhibited significant binding with CDK20 while later AI based optimization produced ISM042-2–048 with a reported binding in the sub micromolar and nanomolar inhibitory range. The compound also demonstrated antiproliferative activity in Huh7 HCC cells with CDK20 overexpression compared with HEK293 cells (47).

### 3.2 Drug design

To build a therapeutic molecule specifically targeting a disease, it is necessary to predict its molecular structure. When the design of the molecule is in contour with the chemical environment of the target protein position, AI aids in drug structure discovery by forecasting the 3D protein structure using tools such as AlphaFold (48). This was done by computing the distance between neighbouring amino acids and the corresponding angles of the peptide bonds. AlphaFold models reliably provide very accurate structural hypothesis for several proteins and biomolecular complexes, but the accuracy of prediction is dependent on target and context and make it essential to validate critical experimentally determined drug structures (49).

Development of AlphaFold2 has blessed AI-driven drug discovery several folds, which evolved from predicting protein structures from amino-acid sequences to structural prediction of complexes including proteins, nucleic acids, small molecules, ions and other biomolecular components in AlphaFold 3 (AF3) (50).

AF3 can produce premises regarding protein–ligand relations, protein–protein edges and protein–nucleic-acid complexes, thus providing structural openings for docking, virtual screening and molecular design. However, one of the drawbacks AF3 is that it predicts structural snapshots rather than ensemble of conformational states altogether populated by a biomolecule in solution, generating structurally reliable complexes even with missing biological relevance essential for inhibitor or activator recognition, which is otherwise important in several clinically relevant targets including Kinases, GPCRs, transcriptional regulators and protein-degradation machinery which can experience structural transitions induced by ligands (49, 50).

AF3 is well suited to relatively static protein–ligand complexes but lacs reliability when binding of ligands are accompanied with larger conformational rearrangements. A study in 2025 reported challenges with complexes concerning conformational changes larger than 5 Å RMSD, with limited reliability for affinity ranking because of its tendency to predict active GPCR- like conformations. Since confidence metrics of AF3 describe structural predictions and do not validate the thermodynamic binding free energy it is less reliable for experimental affinity quantifications or physics-based free-energy methods (49).

The predictive effect of a compound on the target and safety considerations can also be assessed before its synthesis and production. With AI, new drugs with the desired characteristics, such as a high affinity towards the target protein and the least adverse effects, can be developed. The structure of already available medications can be optimized using AI to increase their efficacy and selectivity. Broadly, there are several approaches through which AI is applied in medication development, including the following:

#### 3.2.1 Structure-based drug design

AI can be used to create novel drug compounds that perfectly match the binding pocket of the target protein. This is known as a structure-based drug design. This is accomplished by first developing pharmacological molecules that will attach to the protein in a stable and precise manner, using AI to generate a three-dimensional target protein structure (51).

#### 3.2.2 Ligand-based drug design

Another application of AI is designing novel therapeutic compounds based on the structure of existing medications that bind to target proteins. This is accomplished by first utilizing AI to pinpoint the essential characteristics of existing medications that cause them to attach to the desired protein, and then creating new drug molecules with the same essential characteristics (52).

#### 3.2.3 *De novo* drug design

The potential of *de novo* drug design has been substantially expanded by advancements in deep learning and generative AI models, permitting computational generation and multi-parameter optimization of novel molecular assemblies. In addition, computer-aided synthesis planning can forecast several possible synthesis paths for millions of structures that can be recommended for synthesis. AI is an effective technology that may completely change the development of drugs (53). AI can expedite and reduce the cost of bringing new pharmaceuticals into the market by enabling researchers to design new drug molecules more efficiently and effectively. In addition to being more effective than conventional approaches, AI-powered drug designs can screen many potential therapeutic compounds concurrently. Based on the neural network (NN) score, ML was used to identify the dominant product. For the *de novo* creation of tiny organic compounds, (46) investigated a DNN model termed the ‘reinforced adversarial neural computer (RANC)’ based on RL. The SMILES strings used to represent molecules were used to train the platform. AI can be used to create original drug compounds. This is accomplished by creating many potential drug compounds using AI, which are then screened for necessary qualities (54).

### 3.3 Drug-drug interactions

When two or more medications interact in a way that modifies their effects, they result in DDIs. Serious adverse effects, diminished effectiveness, and even death can result from DDIs. To discover the patterns and correlations related to DDIs, ML models can be trained on massive datasets of known DDIs. After being trained, ML models are employed to forecast the various probability of DDIs occurring between two or more drugs (**Figure 7**). Additionally, DDI severity predictions and individualised DDI risk evaluations for specific patients may be created using AI (55).

**Figure 7:**
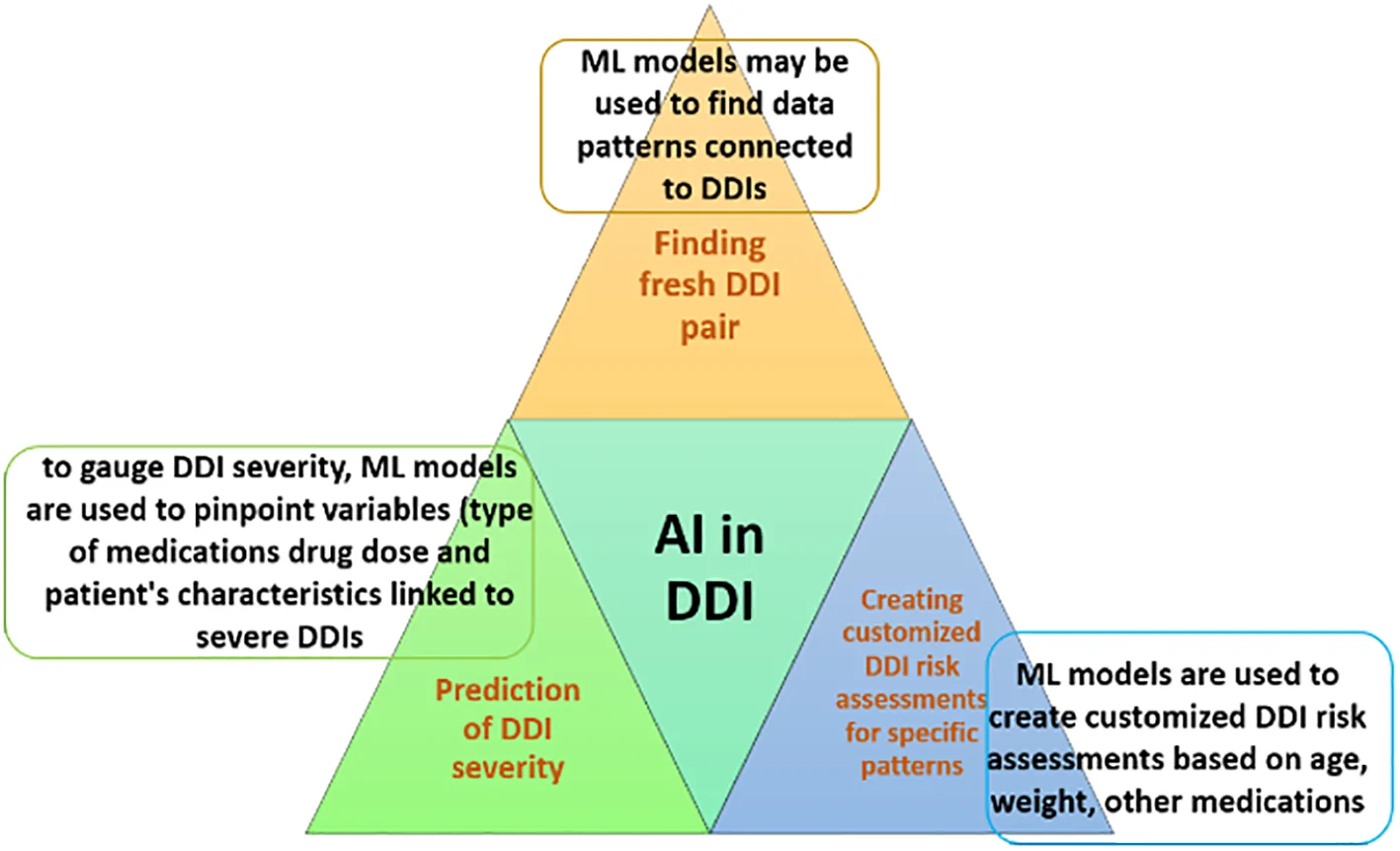
Implementation of AI in drug-drug interactions.

In DDI research, SVMs are frequently used to forecast the probability that a DDI will occur between two or more medications. Various decision trees are combined to form random forests, an ensemble learning model. To forecast the severity of DDIs, random forests are frequently utilized in DDI research. Gradient boosting machines (GBMs) are a different kind of ensemble learning model with several decision trees. To increase the precision of DDI predictions, GBMs are frequently employed in DDI research (56). In order to forecast DDIs and find brand-new DDI pairings, DL models are frequently utilized in DDI research. ML models may be utilised to enhance clinical decision support systems, which will aid doctors in identifying and managing DDIs. When a patient is given a prescription for a drug known to interact with other prescriptions they are taking, this may be accomplished by utilising ML models to produce alerts and cautions.

Since DL models can extract complicated patterns and correlations from data, they are frequently used to forecast DDIs. DL models can produce more precise predictions since they can be trained on even bigger datasets of known DDIs than ML models. Different DL models have been applied to forecast DDIs. To discover the patterns and correlations related to DDIs, DL models may be trained on huge datasets of known DDI pairings (57). Custom DDI risk assessments using DL models may also be utilised to create DDI risk profiles for specific patients. The patient’s unique features, such as age, weight, renal function, and other drugs they are taking, may be considered using DL models. DL models that are specifically being employed in DDI research are illustrated by the following examples such as DeepDDI (58), the DL model that can be employed to foretell the probability that a DDI will occur between two or more medications. Even for medications that have not yet been tested for DDIs, DeepDDI can be used to predict DDIs because it is trained on a huge dataset of known DDI pairings. DDI severity may be predicted using the ML model ddiPred. To forecast the severity of DDIs for new DDI pairings, ddiPred is trained on a sizable dataset of DDIs with known severit (56, 59). Genome-wide association studies (GWAS) use the ML model to find genetic variations connected to DDIs from a sizable dataset of genetic variations. DDI traits are used to train the GWAS model of DDIs.

### 3.4 Assay development

The assay development stage involves designing and optimizing of the assay. AI may be used to create more precise, repeatable, and sensitive assays for the best reagent and assay condition combinations. AI may also create assays that can concurrently detect several different biomarkers. AI can enhance the performance of currently used tests by determining the ideal reagent concentrations and assay incubation periods. AI may also be used to find strategies for simplifying and lowering the cost of an experiment. AI is used to analyse assay data to find patterns and trends that may be used to find novel drug targets and gauge the effectiveness of therapeutic candidates (60). BenevolentAI (https://www.benevolent.com/) uses a combination of SVMs and decision trees to design assays for Alzheimer’s disease. The AI platform of Inflectis BioScience aids in developing assays to predict the toxicity of candidate drugs in preclinical animal models.

### 3.5 Drug repurposing

Drug repurposing, also known as drug redirecting, is a novel approach to reusing and exploring an approved, failed, or abandoned drug and redirecting it towards a new therapeutic application in the same or another morbidity. AI supports drug repurposing in several ways, including by identifying new candidates for repurposing, forecasting the effectiveness and safety of repurposed medications, and streamlining the process. The ‘Guilt by association’ technique can stimulate a novel relationship between drugs and diseased conditions and can be either knowledge-driven or computationally generated. The ML models like SVM, NN, logistic regression and DL are frequently employed in networks that are model driven. When repurposing a medicine, logistic regression platforms, including PREDICT, SPACE and other ML methods, consider drug-drug and disease-disease similarities, drug-target similarity, chemical structure and gene expression patterns (9, 56). Topotecan, a topoisomerase inhibitor currently in use, has been investigated to anticipate its therapeutic usage using cellular network-based DL technology (deepDTnet). Additionally, it can be utilised to treat multiple sclerosis by blocking a human retinoic acid receptor-related orphan receptor (61). In 2020, University of California, San Francisco researchers employed an AI system to find already available medications that might be used to treat COVID-19. An AI system was employed by Stanford University researchers in 2021 to find a medicine that may be utilised to treat Alzheimer’s disease. In a recent work (62), DNN was applied to repurpose 3C-like protease inhibitors, HIV, SARS-CoV, and other medicines having demonstrated efficacy against these viruses. In this case, the training of the AI platform considered the extended connectivity fingerprint (ECFP), functional-class fingerprints (FCFPs), and an octanol-water partition coefficient (ALogP_count). Based on the findings, it was determined that 13 of the tested medicines might be moved towards further development due to their viral inhibition and cytotoxicity.

Li et al. demonstrated KinomeX, an AI-based online platform that utilises DNNs trained with over 14,000 bioactivity data points based on more than 300 kinases to identify polypharmacological kinases (63). Drug–receptor bonding predicts the chances of polypharmacology that tends a drug molecule to bind to multiple receptors producing off-target adverse effects.

Some of the AI and DL models that are being employed to enhance the procedure of drug repurposing include DeepChem (DL library), PredNet (a DL network) to forecast drug-target interactions, DrugComb (AI platform) to find drug combinations that work better than single medications and PhenotypeNet (AI platform) to determine drugs that are effective against a disease or disease phenotypes (64). Information may be extracted from text-based data, such as scientific publications and clinical trial records, using NLP algorithms. This data may be utilised to find possible patients for drug repurposing, forecast the effectiveness and safety of repurposed medications, and streamline the repurposing procedure. AI algorithms can reduce the chances of false positives and negatives, enhancing the accuracy of predicting candidates for drug repurposing.

## 4 Foundation models in cancer drug discovery

The computational goals of drug design have been revolutionized by the emergence of Generative AI from prediction of predefined molecular properties (which earlier generated SMILES strings or molecular graphs) to the generation and optimization of new molecular structures (like sequences, three-dimensional and multimodal representations etc.). The generated molecules can be further optimized by reinforcement learning against numerous purposes, like target affinity, selectivity, physicochemical properties, synthetic accessibility and predicted toxicity (65).

The capacity to explore chemical space beyond compounds is the primordial advantage of generative AI within the training datasets, which however, does not guarantee the representation of all generated molecule as a viable drug candidate. Generation of molecules must satisfy criteria like validity of their chemistry, stability, synthetic accessibility, cellular activity, target engagement, selectivity, pharmacokinetics, toxicity as well as safety and therefore they should be regarded as candidate proposal instead of candidate validation (66).

Foundation models in biology are increasingly representing the next generation systems, where they are pretrained on large quantities of biological data like sequences, structures, molecular data multimodal datasets and essentially scientific literature and subsequently assisting in the downstream applications. A recent study conducted in 2025 identified over 200 foundation models relevant to Drug discovery, RnD in Pharmaceutical applications and demonstrated rapid expansion since 2022 (10).

### 4.1 AI assisted validation of designed drugs:

#### 4.1.1 Physicochemical characteristics of medicinal compounds to be predicted

Using knowledge of absorption, distribution, metabolism, and excretion (ADME) properties, pharmacological molecules may be created that are more likely to be absorbed, distributed, and cause adverse effects in the body. Molecular descriptors (SMILES strings, potential energy readings, electron density surrounding the molecule, and coordinates of atoms in 3D) are used in drug design algorithms to construct viable compounds using DNN and subsequently forecast their attributes (67). To assess the six physicochemical characteristics of surrounding chemicals, Zang et al. developed an EPI suite, a process for quantitative structure-property relationship (QSPR). Neural Networks based on the ADMET predictor and ALGOPS programs have been used to compute the lipophilicity and solubility of various substances. The compounds’ solubility has been prophesied using DL techniques, including graph-based convolutional neural networks (CNN) and undirected graph recursive neural networks (68, 69).

#### 4.1.2 Predicting the toxicity of drug molecules

Several web-based programs may be used to save costs, including LimTox, pkCSM, admetSAR, and Toxtree (70). An ML method named DeepTox, which determines both static and dynamic properties within the chemical filters (molecular weight and van der Waals volume) of the molecules (70), outstripped all other approaches and was able to precisely predict the toxicity of a molecule based on predetermined 2500 toxicophore characters. Using this knowledge, safer drug molecules can be created by patients.

#### 4.1.3 Prediction of drug molecule efficacy

Designing drug molecules more likely to treat a certain condition successfully can be achieved using the knowledge associated with pharmacological molecules (70).

#### 4.1.4 Prediction of bioactivity

The binding capacity of a drug to its target receptor determines its efficacy. The therapeutic effect cannot be produced by drug molecules that do not interact with or have affinity for the target protein. In contrast, similarity-based interactions deliberate the comparation between the drug and target, assuming that they are comparable compounds that interact with similar targets. ML models are used to discover the features of novel therapeutic compounds, such as their propensity to bind to a target protein or their toxicity, which may be predicted using these models. Artificial Neural networks (ANNs) used in DL models can discover intricate patterns and correlations from very large datasets. GNNs are a subset of DL models created to process graph data (71).

### 4.2 Prediction of molecular properties by graphical neural networks

In chemical structures, atoms can be represented as nodes while bonds as edges, and molecular graphs can naturally represent chemical structures. GNNs or graphical neural networks explore this representation via message passing, consenting each atom to update its depiction using information from neighbouring atoms and this feature enables the model to understand chemically meaningful local environments instead of necessitating every relevant structural characteristic to be explicitly encoded with predefined molecular impressions (72). The primary benefit of GNNs over several conventional descriptor-based models is therefore representation learning instead of greater computational complexity. The network learns hierarchical representations from molecular topology, instead of needing the user to specify the essential molecular descriptors. However, comparative benchmarking has established that traditional fingerprints and descriptors can stay competitive, especially when training datasets are small or when the test chemical space varies considerably from the training distribution (73).

#### 4.2.1 AttentiveFP

AttentiveFP incorporates consideration into graph message passing and molecular depiction and thereby assigns different weights to neighbouring atoms and substructures permitting chemically revealing sections of a molecule to donate more strongly to the learned molecular representations. Molecular property prediction utilizes such attention-based representations providing an interpretable initiation point to detect the substructures associated with predicted activity (73).

#### 4.2.2 HiGNN

The Hierarchical Informative Graph Neural Network (HiGNN) encompasses this notion by integrating hierarchical molecular statistics and feature-wise consideration. This model incorporates the chemically expressive molecular fractions using BRICS-based representations, as well as recalibrating atomic feature instead of considering the molecule exclusively as a flat graph. Recent studies demonstrated reliable performance around multiple drug-discovery-related molecular-property datasets, while fractional analysis generated an explainable interpretation of the molecular sections influencing predictions (74).

#### 4.2.3 GNNs for ADMET prediction

The prediction of ADMET stays one of the most essential aspects of GNN guided drug repurposing since the failure of this can give rise to in appropriate absorption, distribution, metabolism, excretion or toxicity related results, eliminating the drug candidates after substantial investment in discovery and development. Thus GNN-based models enable us with early filtering of compounds by learning molecular regional anatomy and endpoint-specific associations before synthesis or expensive experimental characterization (34). Although, GNN approaches have been employed to predict multiple ADMET properties directly using SMILES representations, this predictive accuracy solely is not sufficient for translational deployment. Since a model may generate numerically accurate average predictions as well as being poorly optimized for chemically novel compounds, ambiguous quantification and out-of-domain recognition should be the standard components of AI-assisted ADMET workflows (75, 76).

#### 4.2.4 Geometry-aware GNNs

Geometric deep-learning methods integrate distances, angles, coordinates or symmetry constrictions and therefore deliver a more physically well-versed depiction of molecular structure. Incorporation of molecular three-dimensional geometry into graph-based elucidations is a bench mark as well as conventional 2D molecular graphs encoding connectivity rather than directly describing spatial arrangements stereochemistry, conformational states or non-covalent interactions (77).

In cancer drug detection, ADMET prophecy is predominantly significant as molecular potency solely does not ensure a clinically suitable therapeutic index. AI-based ADMET models can consequently be combined as early multi-parameter filters along with potency and selectivity, allowing candidates with unacceptable toxicity, metabolic instability or pharmacokinetic characteristics to be deprioritized before experimental progression (78).

### 4.3 Other neural networks

To predict the interactions between drug and target, web programs like ChemMapper and the similarity ensemble method (SEA) are available. Many ML and DL-based techniques, including KronRLS, SimBoost, DeepDTA, and PADME, have been applied to predict the DTBA. Kronecker-regularized least squares (KronRLS) evaluate similarity of drug-receptor. SimBoost, on contrary, applies regression trees to compute the DTBA and considers interactions based on feature and similarity. SMILES, ligand maximal common substructure (LMCS), extended connectivity fingerprints, or a combination of these properties can also be studied (79). DL approaches for measuring the DTBA are DeepDTA, PADME, WideDTA, and DeepAffinity. DeepDTA uses drug information in the format of SMILES, in which the list of amino acids for protein input data and the 1D representation of the drug structure are entered. WideDTA is a convolutional neural network (CVNN) approach that assesses binding affinity using ‘ligand SMILES’ (LS), amino acid sequences, LMCS, and protein domains and motifs as input data (80, 81).

### 4.4 Cancer-specific studies: from computational hypothesis to experimental validation

#### 4.4.1 AI-assisted CDK20 inhibitor discovery in hepatocellular carcinoma

The identification of CDK20 as a potential therapeutic target in hepatocellular carcinoma is a benchmark in AI-assisted cancer drug discovery in which a unified pipeline was used for prioritization of target with disease associated molecular data, followed by AlphaFold assisted prediction of structural information as well as Chemistry42 for designing generative compounds. This resulted in the generation of over 8900 candidate molecules of which seven were selected for syntheses and experimental testing followed by optimization of ISM042-2–048 with substantially enhanced biochemical activity with quantifiable CDK20 binding as well as antiproliferative activity in Huh7 cells with overexpressed CDK20 as compared with HEK293 cells (48). This study reliably demonstrates how AI can reduce the quantity of compounds needed for physical evaluation while maintaining experimentally anchored discovery process and represents a preclinical proof of concept relative to evidence of clinical efficacy.

#### 4.4.2 AI-enabled discovery of ENPP1 inhibition for solid-tumor immunotherapy

The convergence of AI assisted prioritization of target, multi-omics investigation and generative chemistry in cancer immunotherapy is well described by a recent example, where ENPP1 was acknowledged as an immune checkpoint regulating extracellular cGAMP and the cGAS–STING pathway followed by designing and optimization of orally bioavailable ENPP1 inhibitor ISM5939. In preclinical models of tumour, ISM5939 improved STING-associated immune activity and presented synergetic effects when administered with PD-1/PD-L1 therapy and chemotherapy (82). Although this illustrates how AI shifted solely from chemical structure optimization to targeted tumour drug therapy, the evidence remains preclinical with first human phase study in advanced metastatic solid tumours in 2025-26.

#### 4.4.3 AI-designed USP1 inhibitor XL309/ISM3091:

The USP1 inhibitor, XL309 (previously denoted ISM3091) is another example of targeted drug therapy in oncology, which was administered in human phase study both as monotherapy as well as in combination with olaparib in patients with solid tumours even though clinical efficacy data are insufficient to establish therapeutic benefit (83).

#### 4.4.4 AI-designed pan-TEAD inhibitor ISM6331

Another instance is a study conducted in 2026 which discusses about ISM6331, an AI-designed pan-TEAD inhibitor synthesized using generative chemistry and structure-based design. The compound targets TEAD-dependent Hippo pathway signalling and has entered clinical development in solid tumours. In August 2026, ISM6331 received FDA Fast Track designation for previously treated unresectable malignant pleural mesothelioma, representing an emerging example of AI-assisted molecular design progressing beyond preclinical development toward an expedited clinical-development pathway. Nevertheless, Fast Track designation does not establish clinical efficacy and should therefore be interpreted as a regulatory-development milestone rather than evidence of therapeutic success (84).

The table below; **Table 1** mentions a comprehensive list of examples of AI- assisted drug discovery in oncology from recent studies.

**Table 1:** Cancer-specific examples of AI-assisted target discovery, molecular design and clinical translation.

AI application	Cancer/indication	AI approach/platform	Target/candidate	Experimental/clinical evidence	Current translational status*
Target identification; structure prediction; molecular design	Hepatocellular carcinoma (HCC)	PandaOmics; AlphaFold2; Chemistry42; multi-omics target prioritization followed by structure-based generative chemistry	CDK20/ISM042-2-048	AlphaFold2-predicted CDK20 structure was used for structure-based molecular generation. From 8,918 generated molecules, seven were synthesized initially; subsequent optimization produced ISM042-2–048 with nanomolar CDK20 inhibition and selective antiproliferative activity in an HCC cell model	Preclinical; validated hit/lead-stage example (48)
Target discovery; generative chemistry	Multiple solid tumors	Patient-derived multi-omics; AI target prioritization; generative AI molecular design	ENPP1/ISM5939	AI and patient-based multi-omics identified ENPP1 as an immunoregulatory target; generative chemistry produced an orally bioavailable ENPP1 inhibitor. ISM5939 showed antitumor activity in multiple syngeneic models and synergized with PD-1/PD-L1 blockade and chemotherapy	Phase I planned/registered; not yet recruiting as of the latest 2026 registry update (82)
AI-assisted molecular design	HRD/BRCA-mutated solid tumors	Generative AI-assisted drug design; Chemistry42/Insilico platform	USP1/ISM3091 (XL309)	Potent, selective and orally bioavailable USP1 inhibitor; preclinical studies demonstrated activity as monotherapy and in combination with PARP inhibitors. The program was subsequently licensed to Exelixis	Phase I; clinical development ongoing (85, 86)
AI-assisted molecular design; target prioritization	MTAP-deleted solid tumors	Generative AI/Chemistry42; AI-supported target and molecule development	MAT2A/ISM3412	Potent and selective MAT2A inhibitor developed for MTAP-deleted cancers; preclinical studies demonstrated pharmacological activity and favorable developability characteristics	Phase I/II; recruiting (87)
AI-driven molecular design	Hippo-pathway-dysregulated solid tumors, particularly mesothelioma	Generative AI/Chemistry42; structure-based design	Pan-TEAD/ISM6331	Demonstrated antitumor activity in NF2-deficient and LATS1/2-mutant mesothelioma models, including activity in combination with EGFR and KRAS-G12C inhibitors	Phase I; recruiting; FDA Fast Track designation in 2026 (84)
AI-driven target identification; molecular design	Advanced solid tumors and B-cell malignancies	AI-enabled target discovery and generative chemistry	QPCTL/ISM8207	Preclinical studies demonstrated antitumor activity across solid-tumor and hematological malignancy models and potential synergy with immune-directed therapies	Phase I; recruiting (88)
AI-driven target/compound discovery; patient stratification	Advanced solid tumors, particularly RCC and NSCLC with high adenosine signaling	Exscientia Centaur Chemist/precision AI design	A2A receptor/EXS21546	AI-assisted design generated a selective A2A receptor antagonist that entered human testing. A subsequent combination study with nivolumab was conducted in advanced solid tumors	Phase I completed; Phase Ib/IIa oncology study terminated in 2023 because of difficulty achieving a suitable therapeutic index (89, 90)
AI-driven molecular design; clinical translation	Advanced solid tumors/transcriptionally dependent cancers	AI-driven molecular design and profiling/Centaur Chemist	CDK7/GTAEXS617 (REC-617)	AI-driven discovery and profiling produced a selective CDK7 inhibitor that progressed from discovery into first-in-human testing	Phase I/II; recruiting (91)
AI-driven drug discovery; generative chemistry	Idiopathic pulmonary fibrosis†	PandaOmics; generative AI/Chemistry42	TNIK/rentosertib (ISM001-055)	First AI-discovered TNIK inhibitor to demonstrate a randomized Phase IIa efficacy signal; the highest-dose group showed increased forced vital capacity relative to placebo	Phase IIa clinical proof-of-concept; non-oncology benchmark (92)

## 5 AI - enabled drug development and clinical translation

Determining the optimum drug dosage is a pivotal step for drug delivery protocol (**Figure 8**). Using QSPR, several computational approaches can be used to overcome challenges with stability, dissolution, porosity, and other aspects of formulation design. The emergence of clinical evidence permitting computational privileges to be assessed against human outcomes is the most important recent development in AI-driven drug discovery. A study published in 2024 discussed the systematic analysis of AI base pipelines which revealed 24 AI generated molecules completed Phase I trials by end of 2023 successfully classifying 21 compounds with 80–90% Phase I success rate which was comparatively higher than the 40–65% rate of historical industry estimates (93). However, one should not completely rely on this interpretation as evidence that AI can efficiently solve clinical drug development as the same analysis demonstrated around 40% Phase II success on smaller dataset.

**Figure 8:**
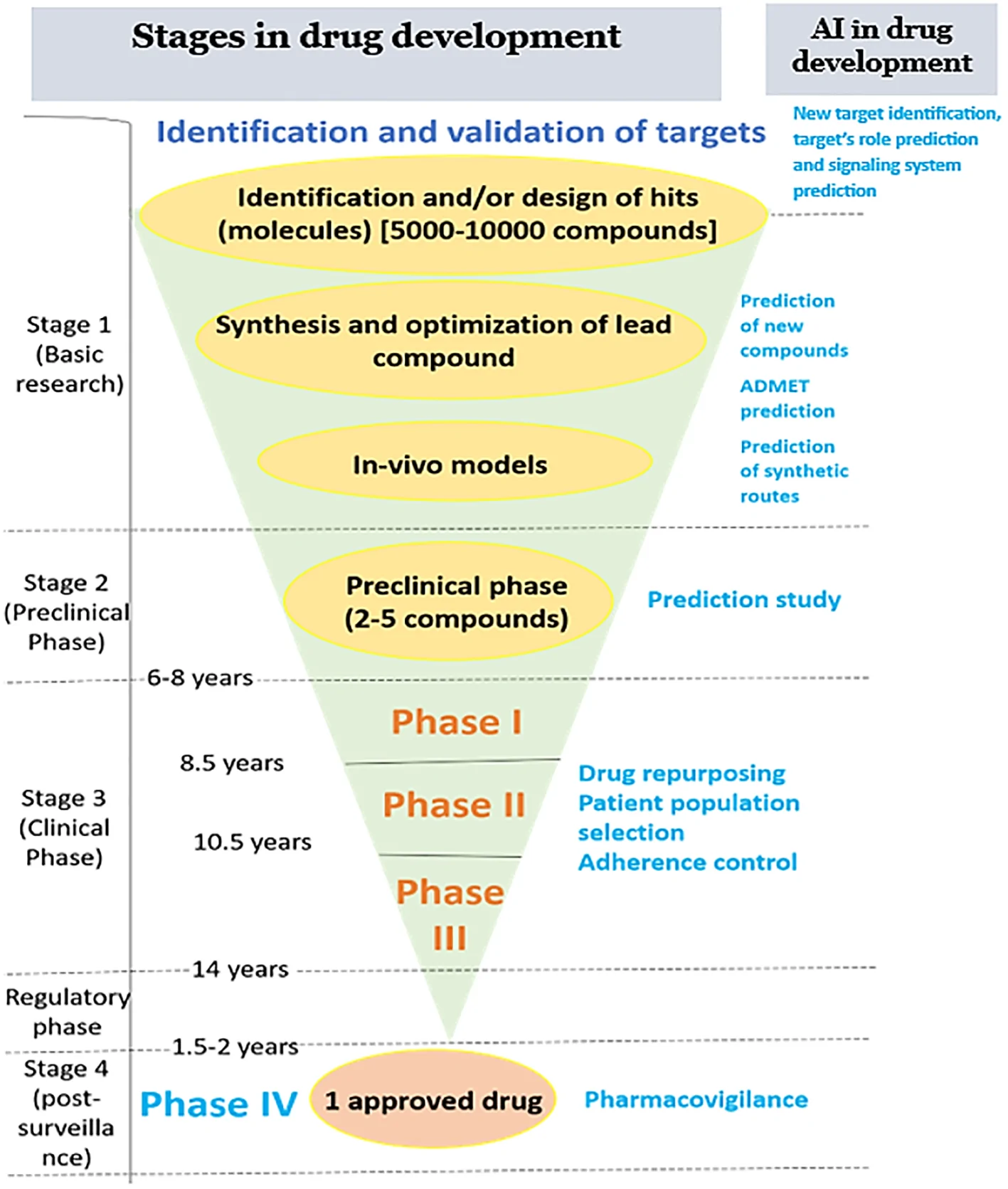
Drug development pipeline showing the application of AI and decision points.

A clinically informative example outside oncology is rentosertib (formerly ISM001-055/INS018_055), an AI-discovered and AI-designed TNIK inhibitor developed for idiopathic pulmonary fibrosis. Subsequently in Phase IIa, 71 patients with idiopathic pulmonary fibrosis across 21 Chinese sites demonstrated rentosertib as well tolerated and established a dose-dependent enhancement in forced vital capacity, providing a significant clinical efficacy signal for an AI-discovered molecule. The study therefore signifies that an AI-generated hypothesis can advance through target identification, molecular design, preclinical development and controlled clinical evaluation (94). Rentosertib, however, not being an anticancer drug, should be rather presented as evidence for wider clinical maturity of AI-driven drug discovery than as evidence of AI accomplishment in oncology.

### 5.1 AI in pre-clinical stages of drug development

One of the most difficult aspects of clinical development is turning **Figure 8**. The Drug development process shows AI’s application at each stage of preclinical discoveries into novel treatments for use in patients (**Figure 9**). Far too frequently, clinical candidates are lost in the translation process. Oncology drug development programmes are the best example of how translational tactics are included as early as possible to raise Phase II and III clinical success rates (95). To do this, clinical development is derisked using translational biomarkers exhibiting target modification, target binding, and confirming proof of mechanism (PoM). Translational biomarker development benefits from the capacity of AI approaches to find hidden and significant motifs by combining huge volumes of heterogeneous and high-dimensional omics data sets. The data collection and the nature of the problem are just two of the numerous variables that influence the decision of the ML algorithm (96) (**Table 2**).

**Figure 9:**
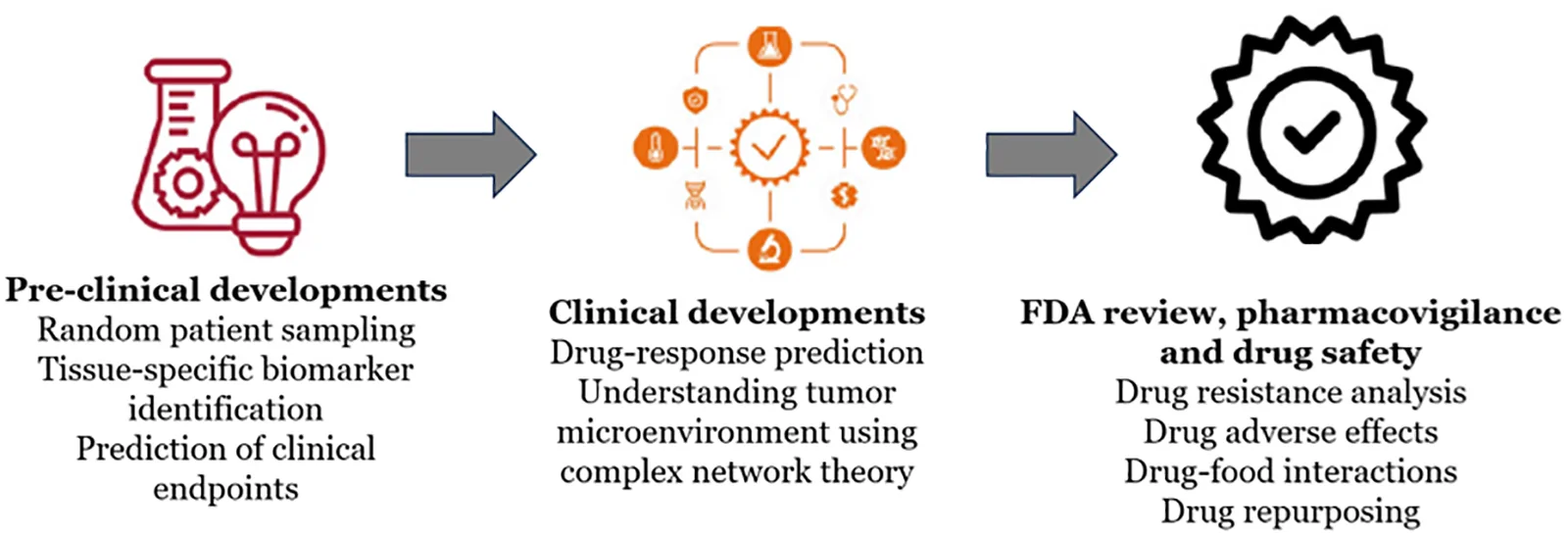
Integration of AI across preclinical development, clinical evaluation, regulatory review and post-marketing pharmacovigilance.

**Table 2:** Comparative evaluation of artificial intelligence methodologies used across the cancer drug-discovery pipeline.

AI approach	Typical representation	Major applications	Main strengths	Major limitations	Translational potential
SVM/RF/GBM	Molecular descriptors/fingerprints	QSAR, toxicity, ADMET, biomarker classification	Performs well on small/medium structured datasets; relatively interpretable (97)	Depends on engineered features;limited representation learning	High for well-defined prediction tasks
CNN	Images/grids/sequences	Histopathology, imaging, molecular representations	Strong spatial pattern recognition (98)	Less natural for graph-structured molecules	High in imaging/pathology
RNN/LSTM	Sequences/SMILES	Molecular generation, sequence modelling	Captures sequential dependencies (99)	Sequential processing; weaker than modern transformers for long-range dependencies	Moderate–high
GNN/MPNN	Molecular graphs	Property prediction, ADMET, DTI	Learns molecular topology directly (100)	Sensitive to dataset quality and chemical-space shift	High
AttentiveFP	Graph, attention	Molecular properties/ADMET	Identifies chemically informative substructures (75)	Attention does not guarantee mechanistic explanation	High
Geometric DL	3D graphs/point clouds	Binding, pose, molecular properties	Incorporates spatial geometry and symmetry (101)	Requires high-quality 3D data; protein flexibility remains difficult	High but requires experimental validation
Generative AI	Molecular graphs/SMILES/3D structures	*De novo* design, optimization	Explores large chemical space (102)	Chemical validity, synthesis, activity and safety remain experimental questions	High for hit/lead generation
Transformer/LLM	Sequences/text/multimodal data	Literature mining, target discovery, molecular generation	Long-range relationships; multimodal integration (103)	Hallucination, provenance and validation problems	Rapidly emerging
Foundation models	Multimodal biological/chemical data	Target discovery, biomarker discovery, molecule optimization	Transfer learning and broad representation (104)	Data leakage, generalization and interpretability	Very high but immature
Reinforcement learning	Sequential decision processes	Multi-parameter molecular optimization	Direct optimization of multiple objectives (105)	Reward hacking and unrealistic chemical solutions	High when constrained by chemistry
Hybrid AI with physics	AI, along with docking/MD/FEP	Structure-based design	Combines data-driven and mechanistic information (106)	Computationally expensive	Very high

### 5.2 Adoption of AI in clinical trials

Understanding and using human data science allows for creating previously impossible insights to obtain solely with healthcare data. Simply put, AI approaches are needed to reconsider how data must be gathered, examined, aggregated, and safeguarded to provide answers, address issues, and make decisions in the healthcare field.

Sponsors may save a significant amount of time and money by working with partners with the required technical and pharmacological experience, lowering risks and raising the calibre of their study. Across many facets of the clinical research process, AI and ML already provide significant time, cost, and quality benefits (95).

#### 5.2.1 Study design

Clinical trials are extremely expensive with a very low chance of success when designed poorly. We can review and choose the best main and secondary outcomes in study design using large healthcare data sets and AI, ML and NLP techniques, ensuring that the most pertinent procedures for regulators, payers, and patients are developed. Providing the best nationwide site strategies, enrolment models, patient recruiting, and start-up plans aids in optimizing the research design. Improved recruitment rates, fewer non-enrolling sites and fewer protocol revisions during research are all benefits of better study design, as are more anticipated results, shorter protocol development cycles, fewer protocol amendments, and greater efficiency (96).

#### 5.2.2 Site finalization and patient recruitment

Studies targeting more specialized demographics face more difficulty recruiting participants, which raises expenses, lengthens study timelines, and increases the possibility of failure. AI and ML may reduce these risks by finding the places with the most potential for recruiting and recommending suitable recruitment techniques. Before a single site is launched, this entails mapping patient demographics, actively seeking places with high estimated potential to deliver the most patients, and determining the best ways to attract them. Sponsors may now create fewer locations, speed up the hiring process, and lower the possibility of under-enrollment (107).

#### 5.2.3 Post-marketing surveillance/pharmacovigilance

To ensure quality and control, structured and unstructured data volumes need to be amalgamated and analysed during PV. Many of the issues that PV units experience in utilising the potential of this data are being addressed by AI and ML technologies, which provide new levels of insight and proactive analytics to improve quality and control. These technologies have the potential to automate highly manual processing workflows and enable the translation and digitization of safety case reports and adverse drug reaction (ADR) records, thereby enhancing their usability and supporting more efficient pharmacovigilance processes (108). They can also execute data listening jobs to monitor talks on social media and other platforms, ensuring that any unfavourable occurrences are noticed as soon as possible. The insights gained by applying AI for PV tasks enable domain experts to analyse subject, site, and study hazards and overall research performance more quickly, allowing project leaders to increase efficiency while enhancing patient safety. For quicker and more effective safety evaluations, optical character recognition (OCR), NLP and DNNs are utilised to analyse and prepare structured and unstructured data. OCR is utilised to auto-identify document types and ingest adverse events (109).

#### 5.2.4 Patient care

Disease detection algorithms are currently being developed to use medical data, such as symptoms and procedures that generally precede a diagnosis, to identify people at high risk of developing a disease. This enables preventative care, novel recruitment insights for prodromal or early-stage illness investigations, and those requiring treatment naïve patients. Because of the exponential consequences of Alzheimer’s disease (AD) diagnostic delays, much clinical research work in AD is focused on the prodromal period. An Electronic Lab Notebook and advanced and intelligent methodologies may provide product quality assurance. Furthermore, data mining and different information discovery techniques combined with quality assessment expert technology are significant to making rigid judgements, resulting in new-edge strategies for better quality control.

#### 5.2.5 Drug delivery and safety assessment

Nanorobots (110) are sophisticated nanoscale devices that integrate components such as sensors, integrated circuits, power sources, and secure data storage, with their operation supported by computational technologies, including artificial intelligence (AI). The development of implantable nanorobots for controlled drug and gene delivery relies on precisely defined parameters, such as dosage regulation and sustained, controlled therapeutic release. Achieving these functions requires intelligent automation, which can be facilitated by AI-based approaches, including neural networks (NNs), fuzzy logic systems, and integrative control algorithms, to optimize delivery accuracy and therapeutic efficacy. AI may also be used to locate drugs with features comparable to those of medications already known to be helpful for a certain condition (111). By examining data from prior clinical trials and other research, AI may be used to forecast the effectiveness and safety of repurposed medications. AI may also be used to find possible medication interactions and negative effects. AI may be utilised to streamline the drug repurposing process. AI may also be used to forecast a drug’s clinical performance, which can assist researchers in choosing which medications to progress into clinical trials.

## 6 Limitations, validation, ethical interpretability and regulatory credibility

AI-driven cancer drug discovery is fundamentally dependent on the quality, completeness and representativeness of the data used for model development and evaluation. Models trained predominantly on well-characterized tumour subtypes, populations or chemical scaffolds may demonstrate strong retrospective performance while generalizing poorly to underrepresented patient populations, novel disease contexts or chemically distinct compounds. Cancer datasets are particularly susceptible to such bias because tumours differ in tissue of origin, molecular subtype, disease stage, ancestry, treatment history and sampling methodology. Independent external validation, prospective evaluation and subgroup-specific performance assessment should therefore be considered essential components of translational AI workflows. (93).

An interrelated methodological concern is outflow of data, where molecules which are structurally similar, inter related protein families, biological replicas as well as patient samples are inadvertently distributed into training and test sets. Random splitting can subsequently yield excessively optimistic estimates of model performance. Scaffold-based, temporal and protein-family based excruciating, followed by sincere external validation, delivers a more rigorous valuation of generalization, predominantly for large underpinning models whose pretraining data may intersect with benchmark datasets (79).

Explainability and mechanical interpretability are correspondingly vital since prognostic precision does not establish organic causality. Attention to visualization, feature attribution methods, SHAP evaluates, counterfactual methods and molecular subgraph investigation can recognize features linked with predictions, but experimental agitation remains essential to validate if an AI-identified target or molecular feature truly initiates the therapeutic phenotype (79).

Progressively, ambiguous quantification should escort molecular forecasts, predominantly for complexes that stay outside the model’s training distribution. Recent GNN-based approaches participating conformal computation can generate standardized prediction interludes for ADMET properties, permitting researchers to discriminate high-performing predictions from those associated with substantial vagueness. Thus, a candidate with comparatively lower predicted potency but considerably greater confidence may be desirable for a highly effective prediction with poorly characterized uncertainty. (79).

Recent clinical studies essentially denote that AI-discovered molecules might have enhanced early drug-like properties without eradicating the fundamental “biology problem”. A study conducted in 2024 reported that around 80–90% AI-discovered molecules gained Phase I success, whereas Phase II success of approximately 40% compared to the historical industry average, which suggests that AI can advance molecular selection and early clinical performance, however the success of its translation yet depends on understanding target dependency, resistance as well as the tumour microenvironment (93).

Another challenge of many commercial AI platforms is their reproducibility which can restrict the access to model architectures and datasets for training them, leading to negative results or reduced performance. Transparent records of datasets, assessment strategies, model limitations and investigational validation are therefore crucial for characterizing genuine technological advances from performance attributable principally to proprietary data or benchmarking practices (93).

Further, the ethical and privacy considerations are also essentially important to prevent irresponsible implementation, inappropriate governance and data handling as well as partial representation and accountability since AI assisted models in cancer biology as well as other domains of health medicine and lifescience incorporates the use of genomic profiles, HER (electronic health records), pathology images and other related clinical data (95).

The regulatory landscape is also evolving simultaneously to protect risk-based credibility assessment of AI-generated content. The U.S. FDA issued guidance drafts which recommended the sponsors to establish a transparent Context of Use (COU) while using AI models demonstrating that the model is satisfactorily reliable for their designated application.

## 7 Future perspectives and conclusion

AI is reshaping the conventional drug discovery techniques by integrating increasingly diverse biological and chemical information by using deep learning, neural networks, LLMs, Generative AI along with other foundation models in biology. Their worth, however, should be mediated by enhancements in biological and clinical decision-making rather than solely by computational scale. Examples such as - CDK20 and ENPP1 inhibitor discovery demonstrate how AI – assisted computational prediction has shaped different aspects of biology like target identification, ADMET prediction, generative molecular design in context of oncology. Clinical translation delivers a more meaningful approach of how AI-discovered molecules have demonstrated reassuring primary clinical performance with success rate of above 80% in phase I stage, pioneering the development of rentosertib on randomized clinical trials. Nevertheless, foremost challenges persist in fundamental target validation, patient assortment, translational modelling and clinical prediction, predominantly because cancer is supported by evolution of tumour, heterogeneity, immune interactions as well as resistance to therapies. Structural AI, such as AlphaFold 3, denotes a foremost advance but necessitates complementary computational and experimental methods because of restrictions in modelling complex conformational states and ligand interactions. Considering the current trends of wide application of AI in biology, it can be assured that the future of therapeutics development will follow a closed-loop human–AI–experiment ecosystem integrating computational prediction, preclinical validation along with clinical insights. It is also possible that the entire microenvironment of the cell or body could be simulated and the activity of a compound can be monitored in respect to the different physio-chemical, pharmacokinetic, pharmacodynamic and other related attributes that govern the activity and efficacy of a drug molecule or compound. However, the excellence in science and technology cannot be solely relied until the hypothesis is successfully validated an along with all these approaches, the regulatory conducts over the development, deployment and fair use should be strictly monitored.
